# Hot water extract of turmeric (*Curcuma longa*) prevents non-alcoholic steatohepatitis in mice by inhibiting hepatic oxidative stress and inflammation

**DOI:** 10.1017/jns.2018.27

**Published:** 2018-12-27

**Authors:** Ryusei Uchio, Shinji Murosaki, Hiroshi Ichikawa

**Affiliations:** 1 Department of Medical Life Systems, Faculty of Life and Medical Sciences, Doshisha University, 1-3 Tatara Miyakodani, Kyotanabe City, Kyoto 610-0321, Japan; 2 Nihon Pharmaceutical University, Komuro 10281, Ina-machi, Kitaadachi-gun, Saitama 362-0806, Japan

**Keywords:** Turmeric (*Curcuma longa*), Non-alcoholic steatohepatitis, Inflammation, Oxidative stress, Fibrosis, ALT, alanine aminotransferase, AST, aspartate aminotransferase, CCR2, CC motif chemokine receptor 2, COL1A1, α1-chain of type I collagen, GSH, reduced glutathione, GSSG, oxidised glutathione, HSC, hepatic stellate cells, KC, Kupffer cells, LMCD, low-methionine, choline-deficient, MCP-1, monocyte chemoattractant protein-1, NASH, non-alcoholic steatohepatitis, ROS, reactive oxygen species, α-SMA, α-smooth muscle actin, SOD, superoxide dismutase, TBARS, thiobarbituric acid-reactive substances, TGF-β, transforming growth factor-β, TIMP-1, tissue inhibitor of metalloproteinases-1, VCAM-1, vascular cell adhesion molecule-1, WEC, hot water extract of *Curcuma longa*

## Abstract

*Curcuma longa*, also known as turmeric, has long been used as a medicinal herb with various biological effects. A hot water extract of *C. longa* (WEC) has been reported to show antioxidant and anti-inflammatory activity, but its effect on hepatic inflammation is poorly understood. In the present study, to investigate the effect of WEC on non-alcoholic steatohepatitis, C57BL/6J mice were fed a low-methionine, choline-deficient diet with 0·175 % WEC (WEC group) or without WEC (control group) for 6 or 12 weeks. Although hepatic steatosis was similar in the WEC group and the control group, WEC suppressed the elevation of plasma aspartate aminotransferase and alanine aminotransferase, which are markers of hepatocellular damage. Compared with the control group, the WEC group had higher hepatic levels of reduced glutathione and superoxide dismutase, as well as a lower hepatic level of thiobarbituric acid-reactive substances. WEC also reduced hepatic expression of mRNA for inflammatory factors, including TNF-α, IL-1β, IL-6, monocyte chemoattractant protein-1, vascular cell adhesion molecule-1, F4/80 and CC motif chemokine receptor 2. Histological examination revealed that WEC suppressed hepatic recruitment of F4/80^+^ monocytes/macrophages and inhibited hepatic fibrosis. Furthermore, WEC inhibited hepatic expression of mRNA for molecules related to fibrosis, such as transforming growth factor-β, α-smooth muscle actin, type I collagen (α1-chain) and tissue inhibitor of matrix metalloproteinase-1. These findings suggest that dietary intake of WEC prevents the progression of non-alcoholic steatohepatitis by alleviating hepatic oxidative stress and inflammation.

Non-alcoholic fatty liver disease is one of the most common liver diseases, with a spectrum ranging from simple steatosis (non-alcoholic fatty liver) to steatosis with hepatocyte injury, hepatic inflammation and fibrosis (non-alcoholic steatohepatitis: NASH). Oxidative stress and production of inflammatory mediators may contribute to the progression of NASH, potentially leading to cirrhosis, liver failure and hepatocellular carcinoma^(^^1^^)^. Despite attempts to treat NASH with various pharmacological agents, clinically meaningful improvement of outcomes has not been achieved^(^^2^^)^.

Both clinical and animal studies have revealed that oxidative stress and impairment of antioxidant defences contribute to liver injury and the development of fibrosis^(^^2^^)^. Accumulation of lipids in the liver leads to increased oxidation of NEFA in parallel with production of reactive oxygen species (ROS), resulting in reduced hepatic tissue levels of superoxide dismutase (SOD) and reduced glutathione (GSH) combined with insufficient removal of ROS due to antioxidant system overload^(^^3^^)^. As a result, oxidative products of lipids, proteins and DNA induce hepatocyte apoptosis and/or necrosis. Transforming growth factor-β (TGF-β) is released by damaged hepatocytes and activated macrophages, and it activates hepatic stellate cells (HSC)^(^^4^^)^ that play an important role in the development of hepatic fibrosis by secreting collagen^(^^5^^)^.

Resident hepatic macrophages such as Kupffer cells (KC, F4/80^+^CC motif chemokine receptor 2 (CCR2)^−^) and inflammatory monocytes (F4/80^+^CCR2^+^) recruited to the liver from the bone marrow are known to have a key role in the development of NASH^(^^6^^)^. In the setting of excessive hepatic lipid accumulation, NEFA activate KC to produce inflammatory cytokines, including TNF-α, IL-1β and IL-6^(^^7^^,^^8^^)^, which can all induce liver damage^(^^9^^–^^11^^)^. In addition, activated KC produce monocyte chemoattractant protein-1 (MCP-1, also known as CCL2), which attracts CCR2-expressing inflammatory monocytes^(^^12^^)^ that amplify hepatocyte injury, inflammation and fibrosis by also producing inflammatory cytokines and chemokines^(^^13^^,^^14^^)^.

*Curcuma longa*, also known as turmeric, is a member of the Zingiberaceae family that has traditionally been used as a medicinal herb with various effects^(^^15^^)^. Aqueous extracts of *C. longa* have been reported to show antioxidant activity^(^^16^^)^ and anti-inflammatory activity^(^^17^^)^, as well as promoting corneal wound healing^(^^18^^)^ and having an anticancer effect^(^^19^^)^. In addition, it was recently reported that a hot water extract of *C. longa* (WEC) inhibits adhesion of monocytes to endothelial cells and prevents alcohol-induced liver injury in mice by decreasing oxidative stress and inflammatory cytokine production^(^^20^^,^^21^^)^.

In order to investigate the effect of WEC on NASH, we examined hepatic steatosis, cellular injury, oxidative stress, inflammation and fibrosis in mice receiving a low-methionine, choline-deficient (LMCD) diet with or without WEC.

## Materials and methods

### Animals

Specific pathogen-free (SPF) male C57BL/6J mice were purchased from SLC Japan and were acclimatised for 7 d before the experiments on a commercial diet (CE-2; CLEA Japan, Inc.). Throughout the experiments, mice were housed in individual cages and were maintained under SPF conditions in a controlled environment (room temperature: 23 ± 1°C, relative humidity: 55 ± 5 % and 12 h light–dark cycle). Experiments were begun at 6 weeks of age (18–22 g) and were carried out in accordance with the guidelines of the Animal Care and Use Committee of Doshisha University.

### Preparation of hot water extract of *Curcuma longa*

WEC was prepared according to a method described previously^(^^20^^)^. It was provided by Takasago International Corporation^(^^22^^,^^23^^)^, and was stored at 4°C until use. WEC had the following composition (w/w): carbohydrate (66·82 %), protein (7·33 %), fat (0·13 %), bisacurone (0·20 %), methionine (0·046 %) and curcumin (0·016 %). Its moisture content was 4·63 % (w/w), and ash accounted for 20·83 % (w/w).

### Experimental design

C57BL/6J mice were allocated to two groups that were balanced with respect to body weight and plasma levels of aspartate aminotransferase (AST) and alanine aminotransferase (ALT). Both groups were fed an LMCD diet with 0·175 % WEC (WEC group; *n* 7) or without WEC (control group; *n* 7) for 6 weeks or 12 weeks. The LMCD diet was prepared by adding 0·05 % (w/w) l-methionine (Wako) to a methionine- and choline-deficient (MCD) diet containing 15 % (w/w) fat (A06083107M; Research Diets)^(^^24^^,^^25^^)^ (Supplementary Table S1). l-Methionine supplementation was done according to Matsumoto *et al*.^(^^24^^)^ to avoid pronounced weight loss associated with the MCD diet^(^^26^^,^^27^^)^. The size of the experimental groups was determined as follows. Our preliminary study revealed that the mean plasma ALT level was approximately 70 (sd 15) IU/l in C57BL/6J mice after 12 weeks on the LMCD diet. In a murine dietary NASH model, quercetin displayed antioxidant and anti-inflammatory effects, reducing the plasma ALT level by about 35 % *v.* the control group^(^^28^^)^. Based on an expected mean plasma ALT level of 70 (sd 15) IU/l in mice on the LMCD diet and a targeted 35 % reduction of plasma ALT by WEC, a group size of seven was required for this study to achieve a statistical power of 80 % with a type I error of 5 %. Plasma AST and ALT levels were measured after 6 weeks and 12 weeks on the diet. Hepatic histological and immunohistochemical changes were assessed immediately before starting the LMCD diet and after 6 weeks and 12 weeks on the diet, as were hepatic antioxidant activity, lipid peroxide content, inflammatory gene expression and pro-fibrogenic gene expression. These parameters were also measured in mice just before starting the LMCD diet (baseline group; *n* 6). Mice were anaesthetised with isoflurane and blood samples were taken from the inferior vena cava. Then the animals were killed by exsanguination, and their livers were harvested and washed with saline to minimise contamination by blood.

### Measurement of plasma aspartate aminotransferase and alanine aminotransferase levels

Blood samples were centrifuged immediately after collection to obtain plasma. AST and ALT levels were measured with a commercial kit (Transaminase CII-test Wako; Wako) according to the manufacturer's instructions^(^^21^^,^^29^^)^.

### Morphological and immunohistochemical analysis of the liver

Liver tissue was fixed in 10 % (v/v) neutral buffered formalin solution, dehydrated with ethanol, cleared in xylene and embedded in paraffin. Then the paraffin-embedded blocks were cut into sections approximately 5 µm thick. After removal of paraffin with xylene, sections were stained with haematoxylin and eosin (Merck)^(^^30^^)^ for morphological analysis, or were stained with Sirius red (Sigma-Aldrich) and counterstained with fast green (Wako) for determination of the area of fibrosis^(^^31^^)^. Immunohistochemical staining of hepatic monocytes/macrophages was performed by using sections of formalin-fixed, paraffin-embedded liver tissue as described previously^(^^32^^,^^33^^)^. Briefly, after removal of paraffin, sections were incubated with a rat anti-mouse F4/80 monoclonal antibody (Serotec), followed by incubation with a biotinylated rabbit anti-rat IgG antibody (Dako) and peroxidase-conjugated streptavidin (Dako). Then colour was developed with diaminobenzidine tetrahydrochloride (Dojindo) and the sections were counterstained with haematoxylin. Images were acquired with an Olympus DP73 digital camera (Olympus IX-73; Olympus) under an inverted microscope (original magnification, ×140). The F4/80-positive area and Sirius red-positive area were quantified as a percentage of the total tissue area by using cellSens Dimension Olympus 1.15 software (Olympus).

### Measurement of hepatic TAG and total cholesterol content

Liver tissue was homogenised in 0·9 % sodium chloride using a disposable homogeniser (BioMasher II; Nippi). Then the homogenate was sonicated with a Sonifire SLPe 40 (Branson) on ice and centrifuged (10 000 ***g*** for 60 min at 4°C), after which the supernatant fraction was stored at −80°C until use. Extraction of the thawed supernatant fraction was performed by the hexane–isopropyl alcohol (3:2, v/v) method^(^^34^^)^, following which the organic solvents were evaporated under reduced pressure. The hepatic TAG content or total cholesterol content was measured with a TG Test Wako kit or Cholesterol Test Wako kit (Wako), respectively, according to the manufacturer's instructions^(^^35^^)^. Both hepatic TAG and total cholesterol were normalised per g of liver tissue (wet weight).

### Measurement of hepatic superoxide dismutase activity

Liver tissue was homogenised in sucrose buffer (0·25 m-sucrose, 10 mm-tris (hydroxymethyl) aminomethane (Tris) and 1 mm-EDTA, pH 7·40) using a disposable homogeniser. The homogenate was sonicated with a Sonifire SLPe 40 on ice and centrifuged (10 000 ***g*** for 60 min at 4°C), after which the supernatant fraction was stored at −80°C. Total SOD, Cu/Zn-SOD and Mn-SOD activities were measured in the homogenate with a SOD assay kit-WST (Dojindo), according to the manufacturer's directions^(^^21^^,^^36^^)^. One unit (U) of SOD activity was defined as the amount causing 50 % inhibition of the reaction in the assay and hepatic SOD activity was normalised per g of liver tissue (wet weight)^(^^37^^)^. Mn-SOD activity was determined by adding potassium cyanide to completely block Cu/Zn-SOD activity, while Cu/Zn-SOD activity was obtained by subtracting Mn-SOD activity from total SOD activity.

### Measurement of hepatic glutathione content and reduced glutathione:oxidised glutathione ratio

Liver tissue samples were added to 5 % (w/v) 5-sulfosalicyclic acid solution and homogenised with a disposable homogeniser. Then the homogenate was centrifuged at 8000 ***g*** for 10 min at 4°C and the supernatant fraction was diluted with deionised water. Total glutathione and oxidised glutathione (GSSG) were determined in the supernatant fraction with a GSSG/GSH Quantification Kit (Dojindo), according to the manufacturer's directions^(^^21^^,^^38^^)^. Then the GSH content was calculated as the difference between total glutathione and GSSG, and the GSH:GSSG ratio was also calculated. The hepatic GSH content, GSSG content and GSH:GSSG ratio were normalised per g of liver tissue (wet weight)^(^^39^^,^^40^^)^.

### Measurement of hepatic lipid peroxides

Thiobarbituric acid-reactive substances (TBARS) were measured in hepatic tissue as a marker of lipid peroxidation. First, liver tissue samples were added to radioimmunoprecipitation buffer (Cayman Chemical) supplemented with protease inhibitor cocktail (Sigma-Aldrich) and homogenised with a disposable homogeniser. The homogenate was sonicated with a Sonifire SLPe 40 on ice and centrifuged (1600 ***g*** for 10 min at 4°C), after which the supernatant was stored at −80°C. Measurement of TBARS in the supernatant fraction was done with a TBARS assay kit (Cayman Chemical) according to the manufacturer's protocol^(^^21^^,^^41^^)^, and the hepatic TBARS level was normalised per g of liver tissue (wet weight)^(^^28^^,^^40^^)^.

### Assessment of hepatic gene expression

Total RNA was prepared by using an RNeasy Lipid Tissue Mini Kit (Qiagen), after which DNA was removed by on-column DNase digestion with a RNase-free DNase Set (Qiagen) according to the manufacturer's protocol. Then gene expression was investigated by real-time PCR^(^^42^^)^. In brief, synthesis of cDNA and PCR were performed using the Thermal Cycler Dice Real Time System TP800 (Takara) and One Step SYBR PrimeScript™ RT-PCR Kit II (Takara) according to the manufacturer's instructions. All primers were obtained from Fasmac and the primer sequences are summarised in Supplementary Table S2. Data were analysed by the 2^−ΔΔCT^ method^(^^43^^)^ using the second derivative curve of amplification plots with Thermal Cycler Dice Real Time System software (version 5.11B; Takara). Expression of the target genes was normalised by glyceraldehyde 3-phosphate dehydrogenase mRNA expression, which was confirmed to be stable by preliminary analysis.

### Statistical analysis

Differences between two groups were assessed with Student's unpaired *t* test. For comparison between the baseline group and the LMCD diet control group, data were also analysed by one-way ANOVA, followed by the Tukey–Kramer test. All analyses were performed with Statcel 3 software (OMS Publishing). Results are shown as means and standard deviations. A *P* value <0·05 was defined as indicating statistical significance.

## Results

### Effect of hot water extract of *Curcuma longa* on body weight, liver weight and hepatic TAG and cholesterol content in mice on a low-methionine, choline-deficient diet

In contrast to the baseline group, body weight showed a significant decrease in the control group after 6 and 12 weeks on the LMCD diet, while the relative liver weight, hepatic TAG content and hepatic total cholesterol content all showed a significant increase at both times. On the other hand, there were no significant differences of these parameters between the control group and the WEC group (Table 1).

**Table 1.** Effect of hot water extract of *Curcuma longa* (WEC) on body weight, liver weight and hepatic lipid content in mice fed a low-methionine, choline-deficient (LMCD) diet (Mean values and standard deviations)

			Feeding period (weeks)							
			6				12			
	Baseline (*n* 6)		Control (*n* 7)		WEC (*n* 7)		Control (*n* 7)		WEC (*n* 7)	
	Mean	sd	Mean	sd	Mean	sd	Mean	sd	Mean	sd
Body weight (g)	21·3^a^	0·5	18·4^b^	1·0	18·0	1·3	19·4^b^	0·7	19·5	0·9
Liver										
Actual weight (mg)	1146^a^	46	1263^a^	153	1145	96	1367^b^	165	1308	151
Relative weight (mg/g body weight)	5·37^a^	0·24	6·86^b^	0·87	6·41	0·95	7·04^b^	0·75	6·68	0·60
TAG (mg/g liver weight)	6^a^	1	271^b^	32	262	26	267^b^	26	256	39
Total cholesterol (mg/g liver weight)	2·50^a^	0·25	3·88^b^	0·26	3·94	0·32	4·90^c^	0·51	4·93	0·42

^a,b,c^Mean values within a row with unlike superscript letters were significantly different (*P* < 0·05; one-way ANOVA, *post hoc* Tukey–Kramer test).

### Effect of hot water extract of *Curcuma longa* on plasma aspartate aminotransferase and alanine aminotransferase levels in mice on a low-methionine, choline-deficient diet

**Fig. 1.**
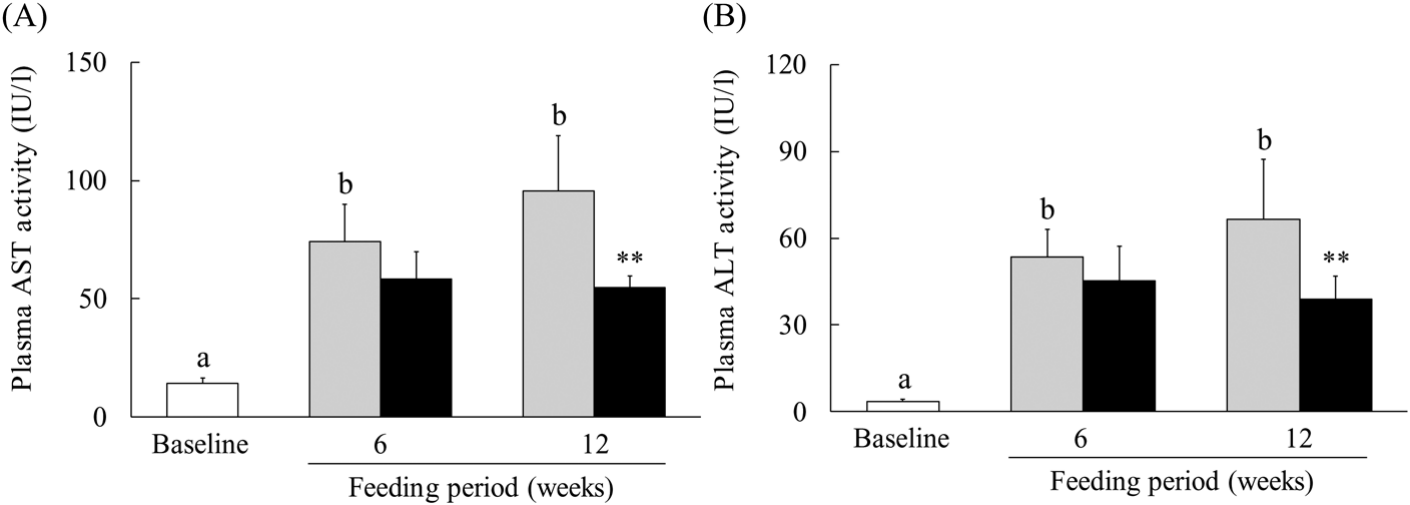
Effect of a hot water extract of *Curcuma longa* (WEC) on plasma levels of liver enzymes in mice. Mice were fed a low-methionine, choline-deficient (LMCD) diet (░) or the same diet supplemented with WEC (■) for 6 or 12 weeks. Plasma levels of aspartate aminotransferase (AST) (A) and alanine aminotransferase (ALT) (B) were measured immediately before starting the LMCD diet (□) and after 6 and 12 weeks on the diet. Data are means (*n* 6: baseline group, *n* 7: control group and WEC group), with standard deviations represented by vertical bars. ^a,b^ Among the bars with letters, mean values with unlike letters were significantly different (*P* < 0·05; one-way ANOVA, *post hoc* Tukey–Kramer test). ** Mean value was significantly different from that of the control group (*P* < 0·01; unpaired Student's *t* test).

Because a WEC has been reported to suppress ethanol-induced liver injury^(^^21^^)^, we evaluated the effect of WEC supplementation on liver damage in mice fed an LMCD diet. In the control group, plasma AST and ALT levels showed significant elevation after 6 and 12 weeks on the LMCD diet, whereas both parameters were significantly lower at 12 weeks in the WEC group compared with the control group (Fig. 1(A) and (B)).

### Effect of hot water extract of *Curcuma longa* on liver histology in mice on a low-methionine, choline-deficient diet

**Fig. 2.**
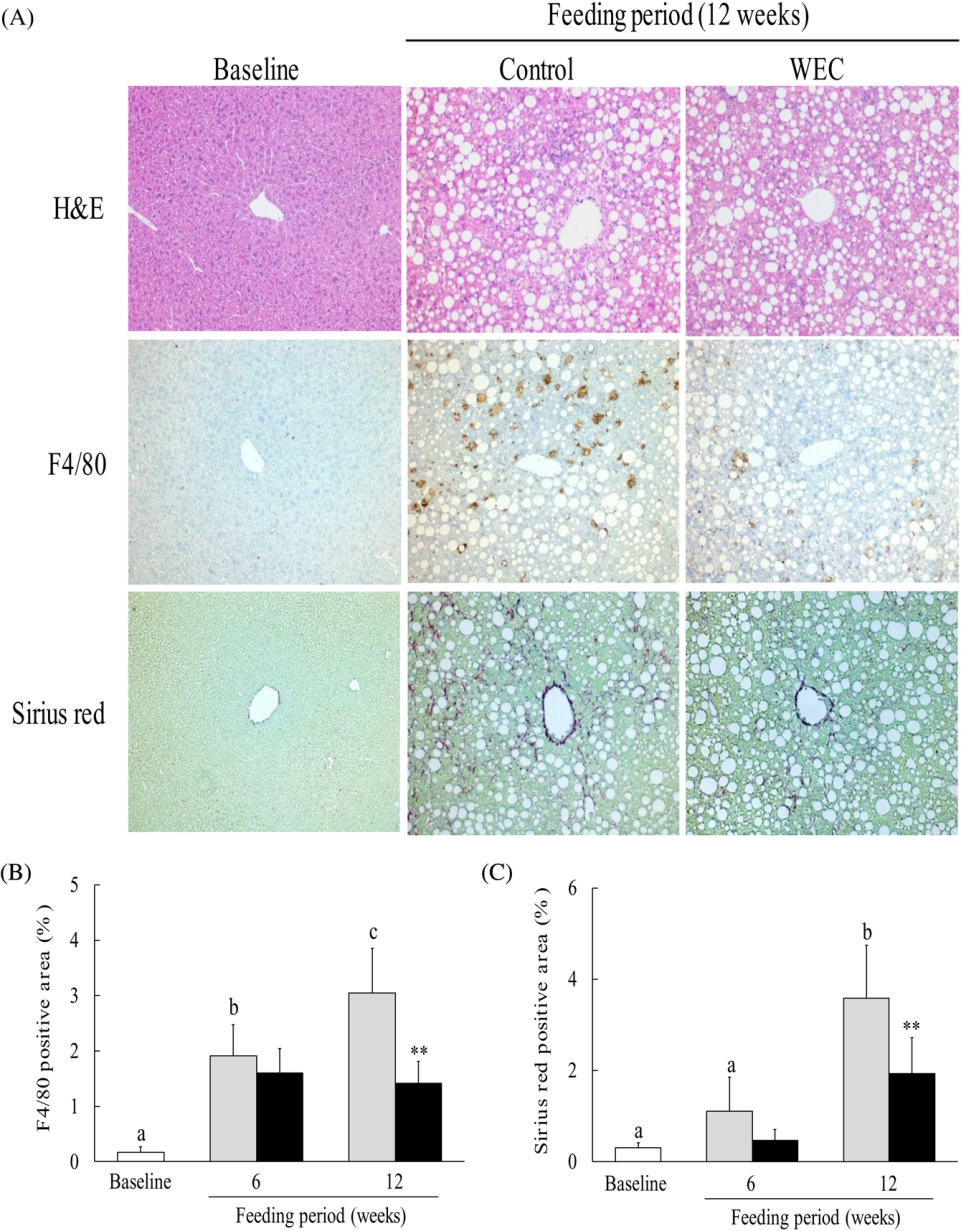
Effect of a hot water extract of *Curcuma longa* (WEC) on liver histology in mice. Mice were fed a low-methionine, choline-deficient (LMCD) diet (░) or the same diet supplemented with WEC (■) for 6 and 12 weeks. (A) Hepatic histology was evaluated by haematoxylin and eosin (H&E) staining (upper), F4/80 staining (middle), or Sirius red staining (lower) before starting the LMCD diet and after 12 weeks on the diet. Original magnification: ×140 for H&E, F4/80 and Sirius red staining. The F4/80-positive area (B) and the Sirius red-positive area (C) were measured before starting the LMCD diet (□) and after 6 weeks and 12 weeks on the diet. Data are means (*n* 6: baseline group, *n* 7: control group and WEC group), with standard deviations represented by vertical bars. ^a,b,c^ Among the bars with letters, mean values with unlike letters were significantly different (*P* < 0·05; one-way ANOVA, *post hoc* Tukey–Kramer test). ** Mean value was significantly different from that of the control group (*P* < 0·01; unpaired Student's *t* test).

The LMCD diet has been reported to cause histological changes consistent with NASH in mice, including hepatic steatosis, inflammation and fibrosis^(^^25^^)^. In this study, we investigated hepatic steatosis and infiltration of F4/80^+^ monocytes/macrophages. In contrast to the baseline group, severe hepatic steatosis was observed in the control group and the WEC group after 6 weeks (data not shown) and 12 weeks (Fig. 2(A)) on the LMCD diet. In the control group, the F4/80-positive area showed a significant increase after both 6 and 12 weeks on the LMCD diet compared with the baseline group, while it was significantly smaller in the WEC group than the control group at 12 weeks (Fig. 2(B)). In addition, the Sirius red-positive area was significantly larger in the control group after 12 weeks on the LMCD diet, whereas it was smaller at 6 weeks in the WEC group compared with the control group (*P* = 0·051) and was significantly smaller in the WEC group at 12 weeks (Fig. 2(C)).

### Effect of hot water extract of *Curcuma longa* on hepatic glutathione content, superoxide dismutase activity and thiobarbituric acid-reactive substances in mice on a low-methionine, choline-deficient diet

To investigate the effect of WEC on antioxidant activity and lipid peroxides in the liver, we measured hepatic total glutathione, GSH, GSSG, GSH:GSSG ratio, Cu/Zn-SOD, Mn-SOD and TBARS. Compared with the baseline group, hepatic total glutathione and GSH were both lower in the control group after 6 and 12 weeks on the LMCD diet, but were significantly higher in the WEC group than in the control group at 6 and 12 weeks (Table 2). In addition, hepatic GSSG was increased in the control group after 12 weeks on the LMCD diet, whereas it was lower at 12 weeks in the WEC group compared with the control group (Table 2). Accordingly, the hepatic GSH:GSSG ratio showed a significant decrease in the control group after 12 weeks on the LMCD diet, while it was elevated at 6 weeks (*P* = 0·056) and significantly higher at 12 weeks in the WEC group compared with the control group (Table 2). Furthermore, hepatic total SOD, Cu/Zn-SOD and Mn-SOD activities all decreased in the control group after 6 and 12 weeks on the LMCD diet, while these enzyme activities were significantly higher in the WEC group at both 6 and 12 weeks compared with the control group (Table 2). Moreover, hepatic TBARS showed a significant increase in the control group after 12 weeks on the LMCD diet, whereas it was lower in the WEC group than in the control group at 12 weeks (Table 2).

**Table 2.** Effect of hot water extract of *Curcuma longa* (WEC) on hepatic antioxidant activities and lipid peroxidation content in mice fed a low-methionine, choline-deficient (LMCD) diet (Mean values and standard deviations)

			Feeding period (weeks)							
			6				12			
	Baseline (*n* 6)		Control (*n* 7)		WEC (*n* 7)		Control (*n* 7)		WEC (*n* 7)	
	Mean	sd	Mean	sd	Mean	sd	Mean	sd	Mean	sd
Liver										
Total glutathione (μmol/g liver weight)	15·6^a^	0·6	12·3^b^	0·9	13·5*	1·1	12·7^b^	1·2	13·9*	0·6
GSH (μmol/g liver weight)	15·4^a^	0·6	11·4^b^	0·8	12·6*	1·1	11·5^b^	1·2	12·9*	0·7
GSSG (μmol/g liver weight)	0·16^a^	0·02	0·15^a^	0·03	0·13	0·02	0·21^b^	0·02	0·18*	0·03
GSH:GSSG ratio	98·1^a^	15·1	76·1^b^	13·7	95·9	20·6	54·9^c^	9·4	75·9*	22·6
Total SOD activity (×10^4^ U/g liver weight)	10·3^a^	0·4	7·8^b^	0·6	10·4**	1·2	7·1^b^	0·8	11·6**	1·4
Cu/Zn-SOD activity (×10^4^ U/g liver weight)	7·59^a^	0·42	6·75^b^	0·66	8·30**	1·09	5·26^c^	0·62	9·35**	1·18
Mn-SOD activity (×10^4^ U/g liver weight)	2·70^a^	0·08	1·03^b^	0·06	2·13**	0·18	1·81^c^	0·24	2·22**	0·21
TBARS (μmol/g liver weight)	27^a^	2	126^a^	110	45	16	240^b^	170	81*	10

GSH, glutathione; GSSG, oxidised glutathione; SOD, superoxide dismutase; TBARS, thiobarbituric acid-reactive substances.

^a,b,c^Mean values within a row with unlike superscript letters were significantly different (*P* < 0·05; one-way ANOVA, *post hoc* Tukey–Kramer test).

Mean value was significantly different from that of the control group: *P < 0·05, ** *P* < 0·01 (unpaired Student's *t* test).

### Effect of hot water extract of *Curcuma longa* on hepatic expression of inflammatory cytokine mRNA in mice on a low-methionine, choline-deficient diet

**Fig. 3.**
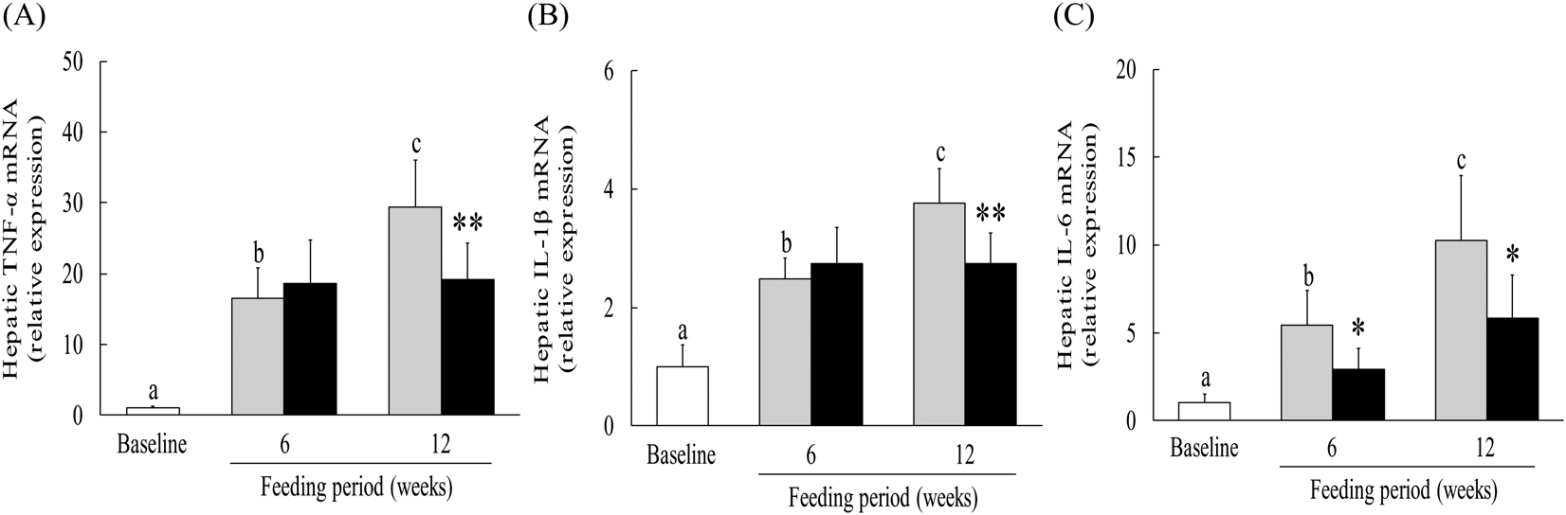
Effect of a hot water extract of *Curcuma longa* (WEC) on hepatic inflammatory cytokines in mice. Mice were fed a low-methionine, choline-deficient (LMCD) diet (░) or the same diet supplemented with WEC (■) for 6 or 12 weeks. Hepatic levels of TNF-α (A), IL-1β (B) and IL-6 (C) mRNA were measured immediately before starting the LMCD diet (□) and while on the diet. Data are means (*n* 6: baseline group, *n* 7: control group and WEC group), with standard deviations represented by vertical bars. ^a,b,c^ Among the bars with letters, mean values with unlike letters were significantly different (*P* < 0·05; one-way ANOVA, *post hoc* Tukey–Kramer test). Mean value was significantly different from that of the control group: * *P* < 0·05, ** *P* < 0·01 (unpaired Student's *t* test).

To assess the effect of WEC on hepatic expression of genes for inflammatory cytokines, we investigated TNF-α, IL-1β and IL-6 mRNA. In the control group, expression of hepatic TNF-α, IL-1β and IL-6 mRNA expression was significantly increased after 6 and 12 weeks on the LMCD diet compared with the baseline group, while TNF-α and IL-1β mRNA expression was significantly lower at 12 weeks in the WEC group than in the control group (Fig. 3(A) and (B)). IL-6 mRNA expression was also significantly lower at 6 and 12 weeks in the WEC group compared with the control group (Fig. 3(C)).

### Effect of hot water extract of *Curcuma longa* on hepatic expression of chemokine, adhesion molecule and inflammatory monocyte marker mRNA in mice on a low-methionine, choline-deficient diet

**Fig. 4.**
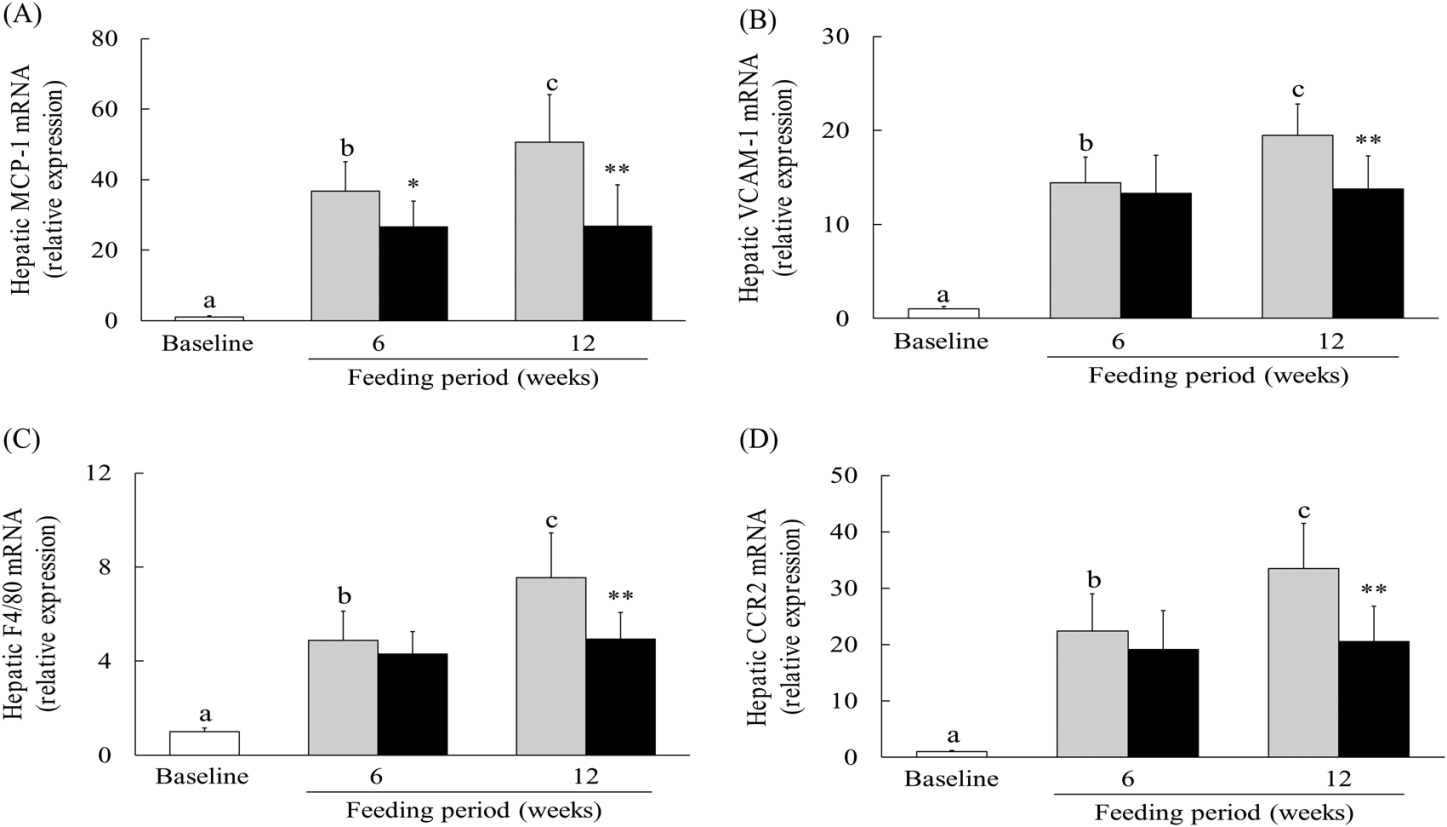
Effect of a hot water extract of *Curcuma longa* (WEC) on hepatic levels of chemokine, leucocyte–endothelial cell adhesion molecule and inflammatory monocyte/macrophage marker mRNA in mice. Mice were fed a low-methionine, choline-deficient (LMCD) diet (░) or the same diet supplemented with WEC (■) for 6 or 12 weeks. Hepatic levels of monocyte chemoattractant protein-1 (MCP-1) (A), vascular cell adhesion molecule-1 (VCAM-1) (B), F4/80 (C) and CC motif chemokine receptor 2 (CCR2) (D) mRNA were measured immediately before starting the LMCD diet (□) and while on the diet. Data are means (*n* 6: baseline group, *n* 7: control group and WEC group), with standard deviations represented by vertical bars. ^a,b,c^ Among the bars with letters, mean values with unlike letters were significantly different (*P* < 0·05; one-way ANOVA, *post hoc* Tukey–Kramer test). Mean value was significantly different from that of the control group: * *P* < 0·05, ** *P* < 0·01 (unpaired Student's *t* test).

To determine the effect of WEC on the expression of chemokines, adhesion molecules and inflammatory monocyte markers, we measured hepatic levels of MCP-1, vascular cell adhesion molecule-1 (VCAM-1), F4/80 and CCR2 mRNA. Compared with the baseline group, MCP-1 and VCAM-1 mRNA expression was significantly up-regulated in the control group after both 6 and 12 weeks on the LMCD diet, while MCP-1 mRNA expression showed significant down-regulation at 6 and 12 weeks in the WEC group relative to the control group (Fig. 4(A)). VCAM-1 mRNA expression was also significantly lower at 12 weeks in the WEC group compared with the control group (Fig. 4(B)). In addition, hepatic expression of F4/80 and CCR2 mRNA showed significant up-regulation in the control group after both 6 and 12 weeks on the LMCD diet, whereas these mRNA were down-regulated at 12 weeks in the WEC group relative to the control group (Fig. 4(C) and (D)).

### Effect of hot water extract of *Curcuma longa* on hepatic expression of pro-fibrogenic mRNA in mice on a low-methionine, choline-deficient diet

**Fig. 5.**
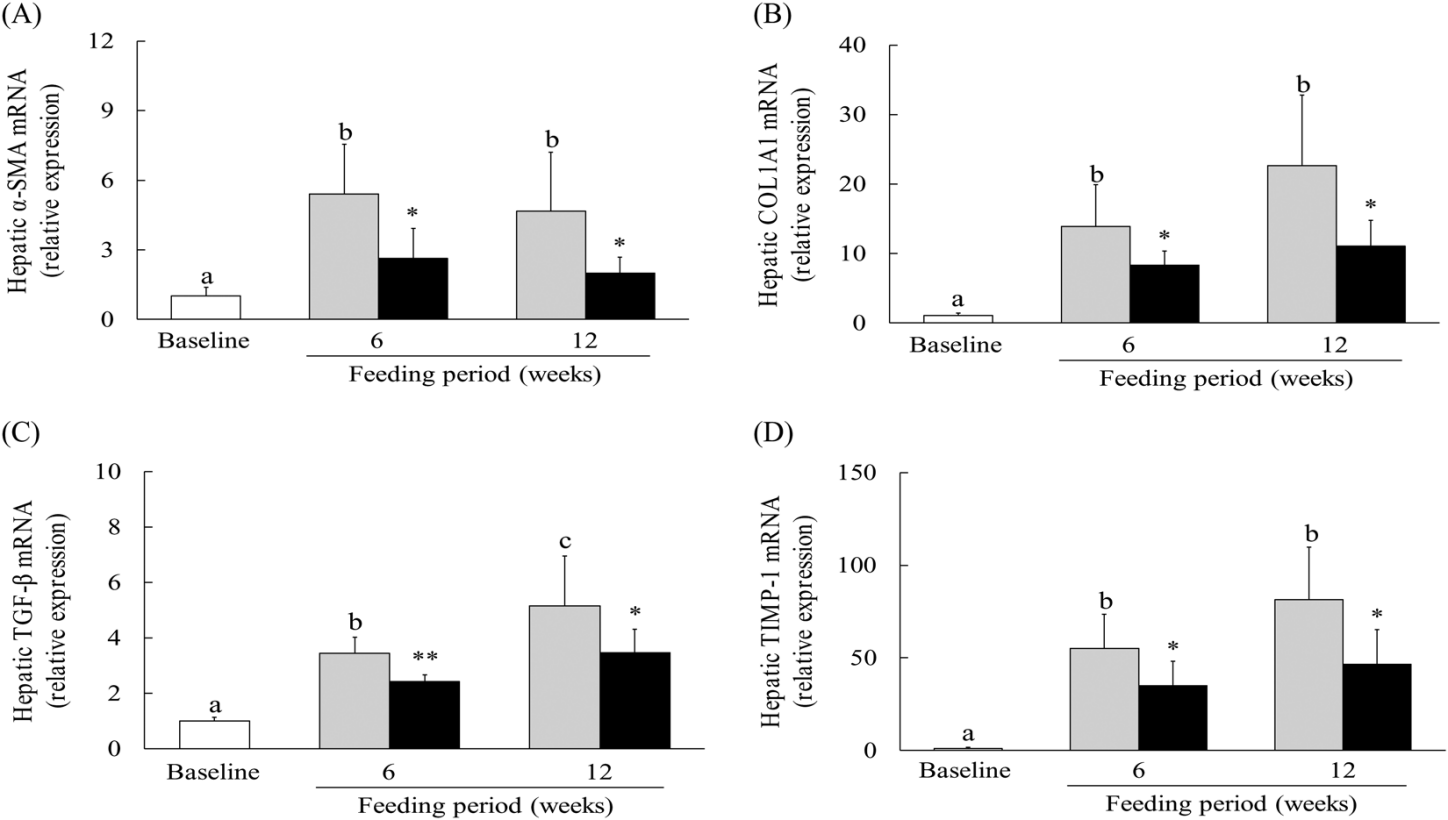
Effect of a hot water extract of *Curcuma longa* (WEC) on hepatic expression of pro-fibrogenic genes in mice. Mice were fed a low-methionine, choline-deficient (LMCD) diet (░) or the same diet supplemented with WEC (■) for 6 or 12 weeks. Hepatic mRNA levels of transforming growth factor-β1 (TGF-β1) (A), α-smooth muscle actin (α-SMA) (B), α1-chain of type I collagen (COL1A1) (C) and tissue inhibitor of metalloproteinases-1 (TIMP-1) (D) were measured immediately before starting the LMCD diet (□) and while on the diet. Data are means (*n* 6: baseline group, *n* 7: control group and WEC group), with standard deviations represented by vertical bars. ^a,b,c^ Among the bars with letters, mean values with unlike letters were significantly different (*P* < 0·05; one-way ANOVA, *post hoc* Tukey–Kramer test). Mean value was significantly different from that of the control group: * *P* < 0·05, ** *P* < 0·01 (unpaired Student's *t* test).

To investigate the effect of WEC intake on hepatic pro-fibrogenic gene expression, we measured the expression of mRNA for TGF-β1, α-smooth muscle actin (α-SMA), α1-chain of type I collagen (COL1A1) and tissue inhibitor of metalloproteinase-1 (TIMP-1). In the control group, expression of TGF-β1, α-SMA, COL1A1 and TIMP-1 mRNA showed significant up-regulation after 6 and 12 weeks on the LMCD diet, whereas expression of these mRNA was significantly down-regulated at 6 and 12 weeks in the WEC group *v.* the control group (Fig. 5(A)–(D)).

## Discussion

The main findings of the present study were as follows. Supplementation with WEC did not affect the liver weight, lipid content, or histological steatosis in mice receiving an LMCD diet. However, WEC significantly suppressed elevation of plasma AST and ALT, a marker of hepatocellular injury, and also reduced hepatic recruitment of F4/80^+^ monocytes/macrophages as well as ameliorating hepatic fibrosis. In addition, the hepatic content of GSH and SOD was significantly higher in mice receiving WEC compared with control mice, while the hepatic TBARS level was lower. Furthermore, WEC significantly inhibited up-regulation of TNF-α, IL-1β and IL-6 mRNA expression in the liver by the LMCD diet, as well as up-regulation of MCP-1, VCAM-1, F4/80 and CCR2 mRNA expression. Finally, expression of pro-fibrogenic genes, including TGF-β1, α-SMA, COL1A1 and TIMP-1, was significantly down-regulated in the WEC group relative to the control group. These results suggest that dietary intake of WEC may prevent progression of hepatic steatosis to NASH by inhibiting oxidative stress and inflammation in the liver.

Although the mechanisms underlying NASH are not well understood, the ‘two-hit’ hypothesis proposed by Day *et al*.^(^^44^^)^ has been widely accepted as explaining its pathogenesis. This theory proposes that genetic factors, drugs and lifestyle factors such as insufficient exercise and high dietary intake of fat cause hepatic accumulation of TAG and NEFA (first hit), after which oxidative stress and inflammatory cytokines (second hit) induce NASH characterised by cellular injury, inflammation and fibrosis^(^^44^^)^. In mice, an LMCD diet has been shown to increase the lipid content, oxidative stress and production of inflammatory cytokines in the liver, as well as provoking histological changes similar to those seen in human NASH, including hepatocellular injury, inflammation and fibrosis^(^^24^^,^^25^^)^. In agreement with previous reports, we found that mice on an LMCD diet showed elevation of the hepatic lipid content, but WEC had no effect on the first hit. However, WEC suppressed hepatic oxidative stress and expression of mRNA for inflammatory factors. Therefore, WEC may prevent the development of NASH, including hepatocellular injury and fibrosis, by inhibiting the second hit.

Increased ROS production and impairment of the antioxidant system secondary to hepatic lipid accumulation may be important in the development of NASH^(^^2^^,^^45^^)^. Normal hepatocyte homoeostasis prevents intracellular accumulation of NEFA and lipotoxic cell damage, but the presence of excess fatty acids can increase ROS production through metabolism of NEFA in the mitochondria and peroxisomes^(^^46^^)^. The subsequent decline of hepatic antioxidant capacity (including GSH and SOD levels) due to excessive ROS production accelerates lipid peroxidation, resulting in apoptosis and/or necrosis of hepatocytes^(^^47^^,^^48^^)^. Additionally, accumulation of cholesterol in hepatocytes can lead to cell death mediated by inflammatory cytokines such as TNF-α or by Fas ligand through mitochondrial GSH depletion^(^^49^^)^. In our previous study, WEC suppressed hepatic oxidative stress induced by acute ethanol administration^(^^21^^)^, with significant amelioration of the reduction in hepatic antioxidant activity as well as inhibition of hepatic lipid peroxidation, as was also noted in the present study (Table 2). These findings indicate that WEC may support hepatic antioxidant activity and suppress the progression of hepatic steatosis to NASH.

NEFA are increased in mice receiving an LMCD diet, and play an important role in the production of IL-6 and MCP-1 by KC, HSC and endothelial cells, resulting in attraction of inflammatory monocytes/macrophages (F4/80^+^CCR2^+^)^(^^50^^–^^53^^)^, while IL-6 further promotes MCP-1 production in an autocrine/paracrine manner^(^^50^^,^^54^^)^. In the present study, WEC significantly inhibited the early up-regulation of hepatic IL-6 and MCP-1 mRNA expression (Figs 3 and 4), as well as significantly suppressing the subsequent hepatic recruitment of monocytes/macrophages (Figs 2 and 4), suggesting that WEC may prevent hepatic infiltration of inflammatory F4/80^+^CCR2^+^ monocytes/macrophages through inhibition of IL-6 and MCP-1 production by KC, HSC and endothelial cells. Inflammatory monocytes/macrophages secrete TNF-α and IL-1β, both of which can directly cause hepatocyte apoptosis/necrosis^(^^9^^,^^10^^)^, and also induce production of leucocyte–endothelial cell adhesion associated protein such as VCAM-1 by endothelial cells^(^^20^^,^^55^^)^. In previous studies, we have shown that WEC not only inhibits hepatic TNF-α production in ethanol-treated mice, but also blocks VCAM-1-dependent adhesion of monocytes to TNF-α-stimulated human endothelial cells^(^^20^^,^^21^^)^. Similar to these findings, WEC significantly inhibited the up-regulation of hepatic TNF-α, IL-1β and VCAM-1 mRNA expression in the present study (Figs 3 and 4). These results suggest that the suppressive effect of WEC on hepatic recruitment of inflammatory monocytes/macrophages may reduce the hepatic production of TNF-α and IL-1β, resulting in inhibition of VCAM-1-dependent infiltration of monocytes into the liver.

HSC are activated by inflammation and oxidative stress, and play a key role in the progression of hepatic fibrosis in NASH by producing extracellular matrix proteins such as collagen^(^^14^^)^. It has been reported that IL-6 induces the activation of HSC with elevation of α-SMA expression via the mitogen-activated protein kinase and signal transducer and activator of transcripts-3 signalling pathway^(^^56^^,^^57^^)^. The resulting increase of hepatic ROS and inflammatory cytokines could potentially induce hepatocyte apoptosis and/or necrosis^(^^9^^,^^10^^,^^46^^)^. When apoptotic cells are engulfed by macrophages, production of TGF-β is induced^(^^58^^)^, while necrotic cells with plasma membrane damage release lipid peroxidation products such as malondialdehyde and 4-hydroxynonenal into the extracellular environment^(^^4^^)^, leading to activation of HSC^(^^59^^,^^60^^)^. In the present study, we found that WEC inhibited various HSC activators, such as extracellular lipid peroxidation substances or IL-6 and TGF-β1 mRNA (Table 2, Figs 3 and 5), resulting in down-regulation of mRNA for α-SMA, a marker of HSC activation. Activated HSC promote an increase of collagen by producing COL1A1 and TIMP-1, which prevents collagen degradation via inhibition of matrix metalloproteinases^(^^14^^)^. In this study, WEC prevented up-regulation of hepatic COL1A1 and TIMP-1 mRNA in mice on the LMCD diet (Fig. 5). These findings suggest that WEC suppressed hepatic collagen deposition by inhibiting the activation of HSC.

*C. longa* has various biological effects and it contains a total of 235 compounds, including primary phenolic compounds and terpenoids^(^^61^^)^. Curcumin is a major anti-inflammatory component of *C. longa* and a suppressive effect on NASH was reported in mice fed a diet containing 1 % curcumin^(^^62^^,^^63^^)^. However, the curcumin content of WEC is too low for it to fully explain the effect of WEC seen in the present study. In addition, there have been no reports about improvement of NASH in mice fed the same dose of methionine (<0·0001 % (w/w)) as that in 0·175 % WEC. Therefore, it is unlikely that methionine would be responsible for the improvement of NASH noted in mice receiving WEC in the present study. Some components of an aqueous extract of *C. longa* have been reported to show anti-inflammatory and antioxidant activity^(^^64^^)^. Bisacurone suppresses elevation of ROS, expression of adhesion molecules and activation of NF-κB (a major transcriptional factor that up-regulates inflammatory cytokines and chemokines) in endothelial cells stimulated by TNF-α^(^^17^^,^^65^^)^ and administration of a single dose of bisacurone (60 µg/kg body weight) significantly suppressed ethanol-induced liver injury in mice^(^^21^^)^. The dose of bisacurone administered to mice in the present study was higher than in the previous study^(^^21^^)^, suggesting that the effect of WEC could be at least partly attributable to bisacurone. Recently, turmeronol A and turmeronol B isolated from WEC were shown to suppress the production of inflammatory mediators by activated macrophages, including PGE_2_ and nitric oxide^(^^66^^)^. It may be important to investigate the effects of these WEC components on NASH in the future.

**Fig. 6.**
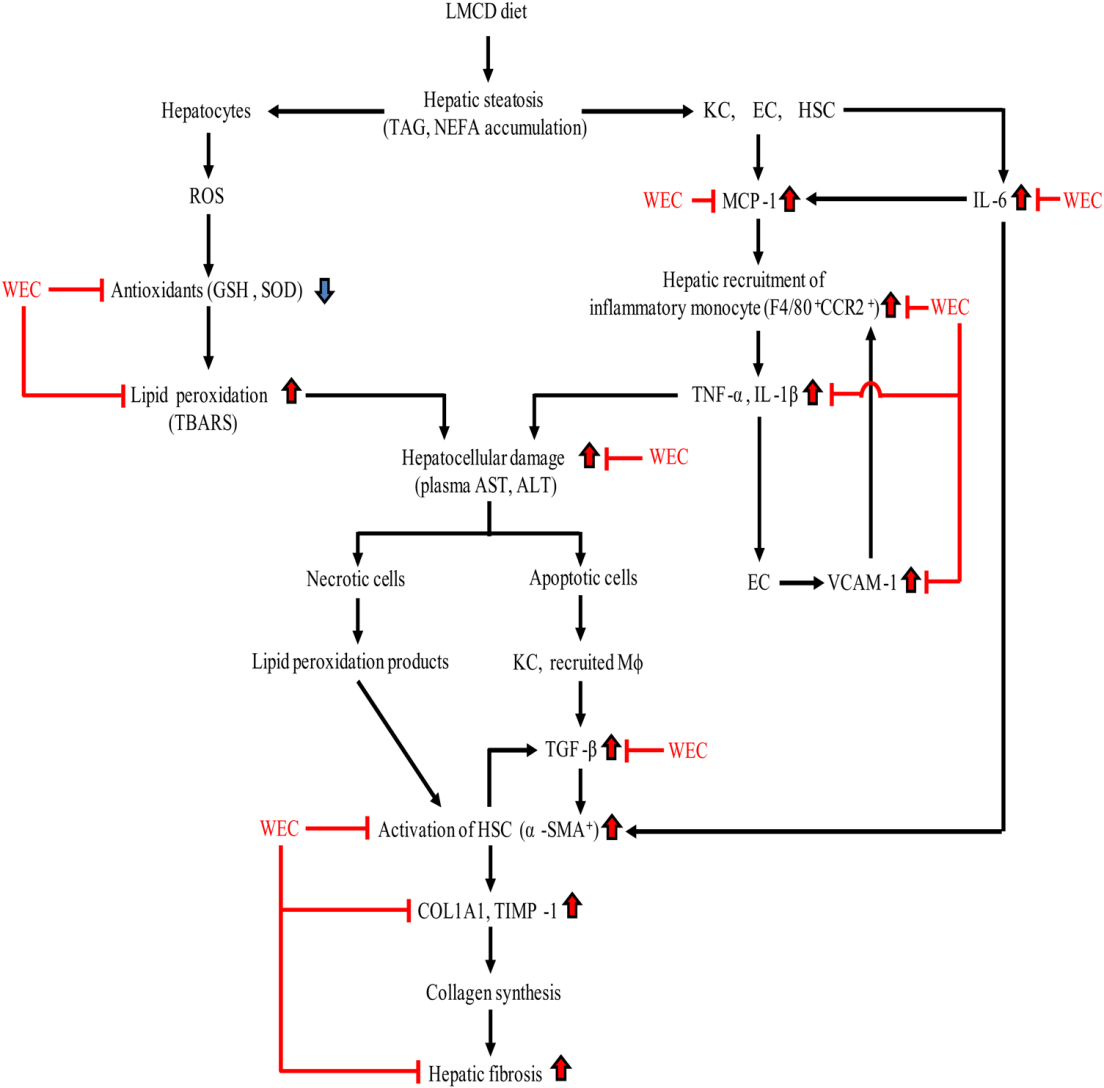
Summary of the effect of a hot water extract of *Curcuma longa* (WEC) on progression of hepatic steatosis to non-alcoholic steatohepatitis. ALT, alanine aminotransferase; AST, aspartate aminotransferase; CCR2, CC motif chemokine receptor 2; COL1A1, α1-chain of type I collagen; EC, endothelial cells; GSH, reduced glutathione; HSC, hepatic stellate cells; KC, Kupffer cells; LMCD, low-methionine, choline-deficient; Mφ, macrophages; MCP-1, monocyte chemoattractant protein-1; ROS, reactive oxygen species; α-SMA, α-smooth muscle actin; SOD, superoxide dismutase; TBARS, thiobarbituric acid-reactive substances; TGF-β, transforming growth factor-β; TIMP-1, tissue inhibitor of metalloproteinases-1; VCAM-1, vascular cell adhesion molecule-1.

In conclusion, WEC supplementation maintained hepatic antioxidant activity in mice on an LMCD diet and suppressed recruitment of inflammatory monocytes/macrophages, thus inhibiting hepatic expression of inflammatory cytokine mRNA and hepatocellular injury. WEC also prevented hepatic fibrosis in the present mouse model of NASH by inhibiting activation of HSC and suppressing collagen production by these cells (Fig. 6). Accordingly, a WEC may have the potential to prevent progression of steatosis to NASH.

## Supplementary material

For supplementary material accompanying this paper visit https://doi.org/10.1017/jns.2018.27. click here to view supplementary material
