# Supplementary material for: Hot water extract of turmeric (Curcuma longa) prevents non-alcoholic steatohepatitis in mice by inhibiting hepatic oxidative stress and inflammation
Source: J Nutr Sci. 2018 Dec 27;7:e36. doi: 10.1017/jns.2018.27 (PMC6313422; doi:10.1017/jns.2018.27)
Supplement: Supplementary file 1 [file S2048679018000277sup.zip › S2048679018000277sup001.doc]

**Supplementary Table S1. Composition of a low-methionine and choline-deficient (LMCD) diet^1^**

| Ingredients | Control group | 0·175% WEC group |
| --- | --- | --- |
|  | g/kg diet | |
| L-Alanine | 5·1 | 5·1 |
| L-Arginine | 12·7 | 12·7 |
| L-Aspartate | 15·8 | 15·8 |
| L-Cystine | 3·7 | 3·7 |
| L-Glutamate | 28·9 | 28·9 |
| Glycine | 6·2 | 6·2 |
| L-Histidine-HCl-H_2_O | 3·4 | 3·4 |
| L-Isoleucine | 6·1 | 6·1 |
| L-Leucine | 10·5 | 10·5 |
| L-Lycine-HCl | 9·1 | 9·1 |
| L-Methionine | 0·5 | 0·5 |
| L-Phenylalanine | 7·3 | 7·3 |
| L-Proline | 7·6 | 7·6 |
| L-Serine | 7·2 | 7·2 |
| L-Threonine | 4·6 | 4·6 |
| L-Tryptophan | 1·8 | 1·8 |
| L-Tyrosine | 5·7 | 5·7 |
| L-Valine | 6·3 | 6·3 |
| Sucrose | 408 | 408 |
| Maize starch | 100 | 100 |
| Maltodextrin | 100 | 100 |
| Cellulose powder | 50 | 50 |
| Maize oil | 50 | 50 |
| Hydrogenated vegetable oil (Primex) | 100 | 100 |
| Mineral mix (AIN76A) | 35 | 35 |
| Sodium bicarbonate | 4 | 4 |
| Vitamin mix (AIN76A) | 10 | 10 |
| Ferric citrate | 0·12 | 0·12 |
| Choline bitarate | 0 | 0 |
| Hot water extract of *Curcuma* *longa* (WEC) | 0 | 1·75 |

^1^ The basal diet was based on a methionine- and choline-deficient (MCD) diet, which was supplemented with 0·05% methionine and 0·175% WEC.
