# Supplementary material for: Hot water extract of turmeric (Curcuma longa) prevents non-alcoholic steatohepatitis in mice by inhibiting hepatic oxidative stress and inflammation
Source: J Nutr Sci. 2018 Dec 27;7:e36. doi: 10.1017/jns.2018.27 (PMC6313422; doi:10.1017/jns.2018.27)
Supplement: Supplementary file 1 [file S2048679018000277sup.zip › S2048679018000277sup002.doc]

**Supplementary Table S2. Real-time PCR primers for analysis**

| Gene | Direction | Primer sequence |
| --- | --- | --- |
| *α-SMA* | Forward （5’-> 3’） | GTCCCAGACATCAGGGAGTAA |
|  | Reverse （5’-> 3’） | TCGGATACTTCAGCGTCAGGA |
| *CCR2* | Forward （5’-> 3’） | AGCACATGTGGTGAATCCAA |
|  | Reverse （5’-> 3’） | TGCCATCATAAAGGAGCCA |
| *COL1A1* | Forward （5’-> 3’） | TAGGCCATTGTGTATGCAGC |
|  | Reverse （5’-> 3’） | ACATGTTCAGCTTTGTGGACC |
| *CTGF* | Forward （5’-> 3’） | GGGCCTCTTCTGCGATTTC |
|  | Reverse （5’-> 3’） | ATCCAGGCAAGTGCATTGGTA |
| *F4/80* | Forward （5’-> 3’） | TGACTCACCTTGTGGTCCTAA |
|  | Reverse （5’-> 3’） | CTTCCCAGAATCCAGTCTTTCC |
| *GAPDH* | Forward （5’-> 3’） | TGTGTCCGTCGTGGATCTGA |
|  | Reverse （5’-> 3’） | TTGCTGTTGAAGTCGCAGGAG |
| *IL-1β* | Forward （5’-> 3’） | TCTTTGAAGTTGACGGACCC |
|  | Reverse （5’-> 3’） | TGAGTGATACTGCCTGCCTG |
| *IL-6* | Forward （5’-> 3’） | CCACTTCACAAGTCGGGAGGCTTA |
|  | Reverse （5’-> 3’） | CCAGTTTGGTAGCATCCATCATTTC |
| *MCP-1* | Forward （5’-> 3’） | GACCCCAAGAAGGAATGGGT |
|  | Reverse （5’-> 3’） | ACCTTAGGGCAGATGCAGTT |
| *TGF-β1* | Forward （5’-> 3’） | CTTCAATACGTCAGACATTCGGG |
|  | Reverse （5’-> 3’） | GTAACGCCAGGAATTGTTGCTA |
| *TIMP-1* | Forward （5’-> 3’） | CTTGGTTCCCTGGCGTACTC |
|  | Reverse （5’-> 3’） | ACCTGATCCGTCCACAAACAG |
| *TNF-α* | Forward （5’-> 3’） | CCTGTAGCCCACGTCGTAG |
|  | Reverse （5’-> 3’） | GGGAGTAGACAAGGTACAACCC |
| *VCAM-1* | Forward （5’-> 3’） | CTACAAGTCTACATCTCTCCCAGGAA |
|  | Reverse （5’-> 3’） | CCTCGCTGGAACAGGTCATT |
